# Formation mechanism and regulation analysis of trumpet leaf in *Ginkgo biloba* L

**DOI:** 10.3389/fpls.2024.1367121

**Published:** 2024-07-17

**Authors:** Xin-hui Li, Xiao-jing Kang, Xin-yue Zhang, Li-ning Su, Xing Bi, Rui-long Wang, Shi-yan Xing, Li-min Sun

**Affiliations:** ^1^ State Forestry and Grassland Administration Key Laboratory of Silviculture in Downstream Areas of the Yellow River, Forestry College of Shandong Agricultural University, Tai’an, Shandong, China; ^2^ Department of Publicity, The Second Affiliated Hospital of Shandong First Medical University, Tai’an, Shandong, China

**Keywords:** *Ginkgo biloba* L., trumpet-type leaf, plant hormone, transcriptome, metabolome, regulatory mechanism

## Abstract

**Introduction:**

The research on plant leaf morphology is of great significance for understanding the development and evolution of plant organ morphology. As a relict plant, the *G. biloba* leaf morphology typically exhibits bifoliate and peltate forms. However, throughout its long evolutionary history, Ginkgo leaves have undergone diverse changes.

**Methods:**

This study focuses on the distinct “trumpet” leaves and normal fan-shaped leaves of *G. biloba* for analysis of their phenotypes, photosynthetic activity, anatomical observations, as well as transcriptomic and metabolomic analyses.

**Results:**

The results showed that trumpet-shaped *G. biloba* leaves have fewer cells, significant morphological differences between dorsal and abaxial epidermal cells, leading to a significantly lower net photosynthetic rate. Additionally, this study found that endogenous plant hormones such as GA, auxin, and JA as well as metabolites such as flavonoids and phenolic acids play roles in the formation of trumpet-shaped *G. biloba* leaves. Moreover, the experiments revealed the regulatory mechanisms of various key biological processes and gene expressions in the trumpet-shaped leaves of *G. biloba*.

**Discussion:**

Differences in the dorsal and abdominal cells of *G. biloba* leaves can cause the leaf to curl, thus reducing the overall photosynthetic efficiency of the leaves. However, the morphology of plant leaves is determined during the primordia leaf stage. In the early stages of leaf development, the shoot apical meristem (SAM) determines the developmental morphology of dicotyledonous plant leaves. This process involves the activity of multiple gene families and small RNAs. The establishment of leaf morphology is complexly regulated by various endogenous hormones, including the effect of auxin on cell walls. Additionally, changes in intracellular ion concentrations, such as fluctuations in Ca^2+^ concentration, also affect cell wall rigidity, thereby influencing leaf growth morphology.

## 1 Introduction

The study of the shape and developmental pathways of plant leaves has consistently been a focal point in botanical research, garnering significant attention in recent years. Leaf morphology stands out as a distinctive feature of plants, showcasing immense diversity and variation (Tsukaya, 2018). Leaves serve as the optimal target for comprehending organ morphology in the context of development and evolution (Lachezar et al., 2019). The types of leaves in plants influence photosynthetic efficiency and environmental adaptability, with their evolutionary trajectory geared towards environmental adaptation. From mosses to ferns and subsequently to seed plants, the evolutionary trend of leaves manifests as a development towards flatness and increased surface area. Nevertheless, the occurrence of leaf curling in certain plant species, contrary to evolutionary trends, has sparked widespread attention.

The application of high doses of herbicides induces leaf edge curling in peanut (*Arachis hypogaea* L.) (Radwan and Fayez, 2016), and plant hormones, especially auxin, are involved in the growth of leaf curl in Wucai (*Brassica campestris* L.) (Hou et al., 2023). Genes *GhARF16-1* and *GhKNOX2-1* in cotton (*Gossypium hirsutum* L.) may serve as potential regulators of leaf morphology (He et al., 2021). The expression of the *ROC8* gene in rice (*Oryza sativa* L.) plants decreases the number and volume of bulliform cells, causing adaxial leaf curling, while knocking out *ROC8* results in abaxial leaf curling (Xu et al., 2021). Moreover, in rice, a transcriptional repression complex comprised of URL1-ROC5-TPL2 was identified, capable of directly binding to the promoter of the positive regulatory factor *ACL1* involved in bulliform cell development and inhibiting its expression, thus regulating the curling of rice leaves (Fang et al., 2021). These studies collectively offer crucial insights into our comprehension of the evolution and molecular mechanisms underlying plant leaf morphology.

*Ginkgo biloba* L. is a deciduous tree belonging to the Gymnospermae division, Ginkgoaceae family, and it falls under the Ginkgo genus. As an ancient plant, *G. biloba* initially exhibited a bifurcated leaf venation, later evolving into *Trichopitys* having hairy leaves with four main veins. In the end, *G. biloba* evolved into the four lobes type, Ginkgoitessp (Zhiyan and Zheng, 2003; Xingwen, 2006; Tang et al., 2022). Despite numerous morphological changes over its long evolutionary history, modern G. biloba still retains ancient dichotomous venation (Beerling et al., 2001). Modern *G. biloba* exhibits various unique leaf forms. Tang et al. conducted anatomical and transcriptomic studies on different *G. biloba* leaf forms, revealing that leaf variation may result from imbalanced near-axis development and leaf margin damage. Genes associated with the axis domain show high expression, and single or double needle leaves may be the outcome of leaf tissue fusion (Tang et al., 2022).

In this study, we conducted a comprehensive analysis of the phenotype, photosynthesis, tissue structure, hormones, metabolomics, and transcriptomics of *G. biloba* trumpet-shaped leaves and normal fan-shaped leaves to comprehend the structural features of *G. biloba* variant leaves. This was followed by the identification of crucial regulatory genes and metabolites associated with *G. biloba* leaf synthesis, and clarification of the regulatory mechanisms governing the formation of distinctive leaf shapes in *G. biloba*. This research not only advances the development and utilization of ornamental *G. biloba* but also establishes a foundation for genetic studies in *G. biloba* after the establishment of the regeneration system. Additionally, it offers valuable insights into the molecular mechanisms of plant morphological variation.

## 2 Materials and methods

### 2.1 Plant materials and growth conditions

The experimental site is located at the Zaoyuan Conservation Repository of the Shandong Forest Germplasm Resource Center in Zhangqiu District, Jinan City, Shandong Province, China. This region is characterized by a temperate continental climate within the warm temperate zone with a mid-latitude. Climatic features include an annual average sunshine duration of 2647.6 hours, with a sunshine rate of 60%; an annual average temperature of 12.8°C, with a high of 13.6°C and a low of 11.7°C; an annual average precipitation of 600.8 mm; and a relative humidity of 65%, with the highest annual average humidity at 73% and the lowest at 59%.

**Figure 1:**
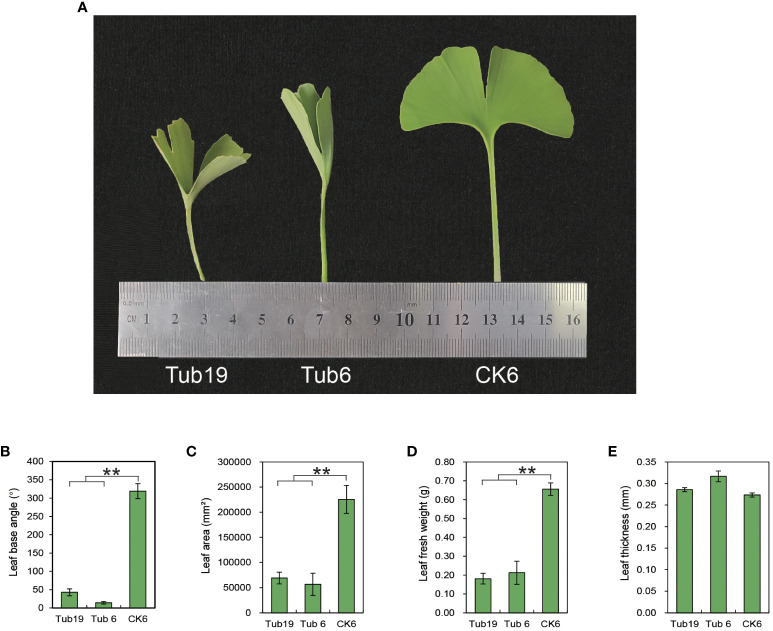
Phenotypes of different test materials. **(A)** Phenotypes of three leaves. **(B)** Differences in leaf basal angles among three types of leaves. **(C)** Differences in leaf area among the three leaf species. **(D)** Difference in fresh leaf weight among the three leaves. **(E)** Differences in blade thickness between the three types of blades. Error bars indicate ± S.E. from three biological replicates (**p < 0.01).

The experimental materials consist of two *G. biloba* clones, named ‘Tubifolia-19’ and ‘Tubifolia-6’, which were selected (**Figure 1A**). We systematically selected plants from each clone that exhibited consistent size, were free from diseases and pests, and demonstrated similar growth conditions. This included trumpet-shaped leaf blades (Tub19 and Tub6), as well as normal leaves on ‘Tubifolia-6’ plants (CK6), serving as the research materials.

### 2.2 Leaf morphology observation and sectioning experiments

Leaf morphological parameters were measured, including length, width, single leaf area, fresh weight, dry weight, relative water content, thickness, and baseline angle of the leaf (**Supplementary Figure S1**).

Leaf section experiments require material selection from the experimental site, followed by processes such as fixation, cleaning, dehydration, permeation, embedding, aggregation, preservation, block and section preparation, staining, microscopy, and paraffin section observation and analysis (Cao et al., 2023).

### 2.3 Determination of leaf photosynthesis parameters

On a sunny day in June 2022, from 9:30 to 11:30, the LI-6800 portable photosynthesis system was utilized. The CO_2_ concentration was set to 800µmol mol^⁻¹^, and the light intensity to 1200µmol m^⁻²^ s^⁻¹^. Measurements included net photosynthetic rates of cylindrical and normal fan-shaped leaves on both sides, as well as various photosynthetic parameters such as transpiration rate, stomatal conductance, and intercellular CO_2_ concentration. Three leaves per leaf type were measured for each experimental group.

### 2.4 Transcript profiling

On a selected day in June 2022, we collected 3 sets of experimental materials for both the transcriptome and metabolome at the experimental site. Each group had three replicate samples, which were promptly stored in liquid nitrogen at -80^°^C to ensure their preservation. The specific transcriptome sequencing and data analysis include ①RNA extraction and library construction. RNA was extracted using Aidlab RN 38, and cDNA was synthesized using the Prime Script II 1st cDNA Synthesis Kit. RNA degradation and contamination were monitored on 1% agarose gels. The NanoPhotometer spectrophotometer (IMPLEN, CA, USA) was utilized to assess RNA purity (OD260/280 and OD260/230 ratios), and RNA concentration was accurately quantified using the Qubit2.0 Fluorometer (Life Technologies, CA, USA). Additionally, RNA integrity was assessed using the Bioanalyzer 2100 system (Agilent Technologies, CA, USA). Sequencing libraries were generated using NEBNextRUltraTMRNA Library Prep Kit for lluminaR (NEB, USA) following the manufacturer’s recommendations, and sequencing was performed on the Illumina sequencing platform (Illumina novaseq 6000, Illumina, USA, Sequencing strategy: PE150), generating paired-end reads of 150/125 bp. The sequence data are available in the NCBI Sequence Read Archive (SRA) under accession numbers SRR18002871, SRR18002870, SRR18002869, SRR18002868, SRR18002867, SRR18002866, SRR18002865, SRR18002864, and SRR1800286. ②Sequencing data filtering and reference genome comparison. We used fastp (0.23.2) to filter the original data, primarily to remove reads with adapters. Paired reads were removed when the N content in any sequencing read exceeded 10% of the total bases. Additionally, paired reads were discarded when the number of low-quality bases (Q<=20) exceeded 50% of the total bases in the reads. All subsequent analyses were based on clean reads. The clean reads were then aligned to the reference genome (https://ngdc.cncb.ac.cn/gwh/Assembly/18742/show) using HISAT (2.2.1, default parameters) (Kim et al., 2015). ③Functional annotation and enrichment analysis of gene expression genes. Normalization of gene expression levels by DESeq_2_ (Love et al., 2014; Varet et al., 2016), as well as analysis of Differentially expressed gene (DEG) data for each sample, were conducted. The screening condition was |log_2_Fold Change|≥1, FDR ≤ 0.05. DEGs were identified through Gene Ontology (GO, 20220720) and Kyoto Encyclopedia of Genes and Genomes (KEGG, 20220720) analyses. GO enrichment and KEGG pathway enrichment analyses of DEGs were conducted using R. Enrichment items corrected to P<0.05 were considered significantly enriched.

### 2.5 Metabolic profiling

Comprehensive metabolomic analysis was conducted by Wuhan Metware Biotechnology Co., Ltd. In summary, biological samples were subjected to freeze-drying using a vacuum freeze dryer (Scientz-100F) and ground with a 30Hz mixer mill (MM 400, Retsch) for 1.5 minutes each. Subsequently, 50 mg was dissolved in 1.2mL of a 70% methanol solution (vortexed for 30 seconds every 30 minutes, totaling six times). After centrifugation at 12000 rpm for 3 minutes, the supernatant was filtered through a 0.22μm pore size membrane and employed for ultra-performance liquid chromatography (UPLC) and tandem mass spectrometry (MS/MS) analysis.

Specific data acquisition was performed using UPLC and MS/MS. The specific workflow of the UPLC-ESI-MS/MS system (UPLC, ExionL C™ AD; MS, Applied Biosystems 4500 Q TRAP) was conducted according to the methods outlined by Yuan et al (Yuan et al., 2022).The ESI source operation parameters were source temperature 550°C and ion spray voltage (IS) 5500 V (positive ion mode)/-4500 V (negative ion mode). The other operating parameters followed the methods of Chen et al. (W. Chen et al., 2013). Triple Tandem Quadrupole Mass Spectrometer (QQQ) scans were acquired as multiple reaction monitoring (MRM) experiments with collision gas (nitrogen) set to medium. DP (declustering potential) and CE (collision energy) for individual MRM transitions was done with further DP and CE optimization. A specific set of MRM transitions was monitored for each period according to the metabolites eluted within this period. Analyst 1.6.3 software was employed for the processing of mass spectrometry data. During the instrument analysis, a quality control sample was inserted for every 10 samples to perform sample quality control analysis.

Qualitative analysis of substances was performed based on secondary spectrum information using the self-built database MWDB (metware database), and metabolites were quantitatively analyzed using MRM with triple quadrupole mass spectrometry. Differential metabolites were screened using parametric tests, non-parametric tests, combined principal component analysis (PCA), and partial least squares discriminant analysis, with screening criteria of VIP≥1 and |Log_2_FC|≥1.0. For specific qualitative and quantitative analysis of experimental samples and the screening of differential metabolites between samples refer to Chen et al. (C. Chen et al., 2022).

### 2.6 Plant endogenous hormone detection

Hormone analysis employed the LC-MS/MS platform. Eight types of plant hormones were detected, including auxin, cytokinins (CKs), abscisic acid (ABA), jasmonic acid (JA), salicylic acid (SA), gibberellin (GA), ethylene (ETH), and strigolactones (SL). The experimental sample handling procedures were referenced from Li et al. (Y. Li et al., 2016). The chromatography-mass spectrometry acquisition conditions were set based on the experiments conducted by Hua, Šimura, and others (Simura et al., 2018; Xiao et al., 2018).

### 2.7 Real-time fluorescence quantitative PCR to verify transcript levels of relevant genes

The methods for sample collection, RNA extraction, and reverse transcription are consistent with those used in the transcriptome analysis. In total, 16 candidate genes associated with *G. biloba* leaf shape formation were selected from three experimental samples for real-time fluorescence quantitative validation to confirm the expression of DEGs in the transcriptome. *GbACT* was used as an internal reference gene and primers were designed using Primer Premier 6.0 (**Supplementary Table S1**). The qRT-PCR reaction system and procedures are provided in **Supplementary Table S2**. All reactions and analyses were conducted using SYBR Premix Ex Taq (TaKaRa) and the Bio-Rad CFX96 PCR system. The Livak method (2^-ΔΔCt^ method) was employed to calculate the relative expression levels. The experiment was performed with three independent biological triplicates, with each triplicate comprising three technical replicates.

### 2.8 Statistical analyses

Data organization and graph creation were executed using Microsoft Excel and GraphPad Prism 9.5 (GraphPad Software Inc., Boston, MA, USA). Physiological data underwent T-test analysis utilizing SPSS 25 (Inc., Chicago, IL, USA). Annotation and categorization of endogenous plant hormones, DAM, and DEG were carried out using the KEGG database (Kanehisa, 2000). Venn diagrams were created using Venn Diagram (1.6.20, default parameters). Heatmaps were generated, and clustering analysis was performed using the ComplexHeatmap package (2.8.0) in R and TB tools. WGCNA analysis plots were performed using the plotMat function (https://rdrr.io/cran/WGCNA/src/R/smaFunctions.R#sym-plotMat) in the WGCNA package (V1.69) in R [version 3.5.1 (2018-07-02)} and the barplot function (https://rdrr.io/github/BillVenables/WWRGraphics/man/barplot.html) in the graphics package (V3.5.1). Before constructing the co-expression network using the R language WGCNA (1.71), it was necessary to filter the FPKM data file related to gene expression. The varFilter function from the genefilter package in R was used to remove genes with low expression across all samples and genes with stable expression across all samples, with a soft threshold of 0.85 selected. The clustering dendrogram was cut at a height of 0.25 to merge dynamic modules with similar patterns (Zhou et al., 2022). Genes in the green module that were highly expressed in trumpet-shaped leaves (Tub19, Tub6) and lowly expressed in normal fan-shaped leaves (CK6), as well as genes in the black module that were lowly expressed in trumpet-shaped leaves (Tub19, Tub6) and highly expressed in normal fan-shaped leaves (CK6), were selected for further analysis (Zhou et al., 2022). DEGs and differential metabolites were mapped to the KEGG pathway database to obtain their shared pathway information. Predict transcription factors using iTAK (http://itak.feilab.net/cgi-bin/itak/index.cgi, 1.7a). Bio-Rad CFX96 software was utilized for the analysis of the qRT-PCR data. The final layout and design were accomplished using Adobe Illustrator (AI).

## 3 Results

### 3.1 Phenotypic observation of *G. biloba* leaves with different leaf shapes

Trumpet-shaped *G. biloba* leaves (Tub19, Tub6) exhibit a non-flat shape with an inward curl, forming a cylindrical structure. Phenotypic data for *G. biloba* leaves with three different shapes were measured and analyzed (**Figure 1A**). The results reveal that the leaf basal angle, leaf area, and leaf fresh weight of trumpet-shaped leaves (Tub19, Tub6) were significantly smaller than those of normal fan-shaped leaves (CK6) (**Figures 1B-D**). However, there was no significant difference in leaf thickness among Tub19, Tub6, and CK6 (**Figure 1E**; **Supplementary Table S3**).

### 3.2 The slice experimental observation of *G. biloba* leaves with different leaf shapes

**Figure 2:**
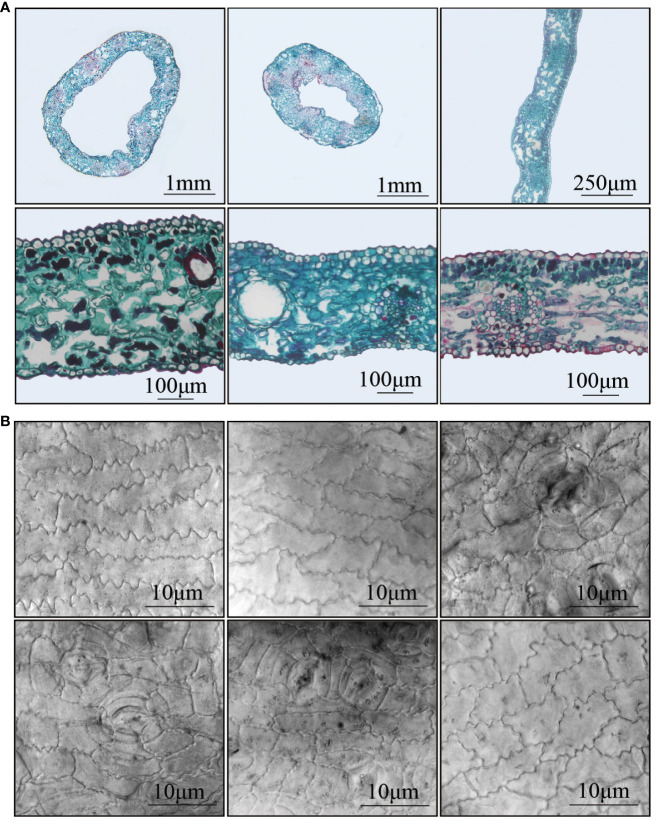
Anatomical diagram of different (*G*) *biloba* leaves and observation of epidermal cells. **(A)** From left to right, from top to bottom: the cross-section of Tub19 blade (annular shape), the cross-section of Tub6 blade (annular shape), the cross-section of CK6 blade (ribbon), the cross-section detail of Tub19 blade, the cross-section detail of Tub6 blade, and the cross-section detail of CK6 blade. **(B)** From left to right, from top to bottom: the abdominal epidermal cells of Tub19 leaves, the abdominal epidermal cells of Tub6 leaves, the abdominal epidermal cells of CK6 leaves, the dorsal epidermal cells of Tub19 leaves, the epidermal cells of the dorsal back of Tub6 leaves, and the epidermal cells of the dorsal of CK6 leaves.

Three types of leaves, located approximately 5cm from the leaf base, were dissected. The cross-section of trumpet-shaped leaves (Tub19, Tub6) appeared ring-like, while normal fan-shaped leaves (CK6) exhibited a strip-like pattern (**Figure 2A**). The inner side (abdominal) of trumpet-shaped leaves (Tub19, Tub6) corresponded to the upper epidermis, and the outer side (dorsal) corresponded to the lower epidermis. Analysis of Specific data (**Supplementary Table S4**) revealed that, regardless of leaf type, the thickness of the single-layer cells in the abdominal epidermis was greater than that in the dorsal epidermis for all samples. Additionally, compared to CK6, the abdominal epidermal cells of trumpet-shaped leaves (Tub19, Tub6) had a larger individual cell area, and the abdominal epidermal cell area was significantly larger than the dorsal epidermal cell area for all three leaf types (**Figure 2B**). Observation showed that both the thickness of single-layer cells and the individual cell area gap between the abdominal and dorsal epidermis of trumpet-shaped leaves (Tub19, Tub6) were much larger than those of normal fan-shaped leaves (CK6) (**Supplementary Table S4**). Moreover, examination of epidermal cells in trumpet-shaped leaves (Tub19, Tub6) and normal fan-shaped leaves (CK6) revealed that the stomata of Tub were mostly located on the dorsal side, while those of CK were situated on the abdominal side of the upper epidermis (**Figure 2B**).

### 3.3 Determination and analysis of photosynthetic indexes of *G. biloba* leaves with different leaf shapes

**Figure 3:**
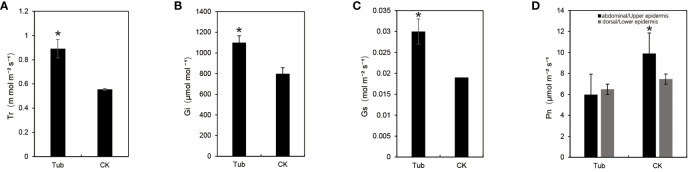
Photosynthetic data for different leaf shapes. **(A)** transpiration rate; **(B)** the intercellular CO_2_ concentration; **(C)** stomata conductance; **(D)** net photosynthetic rate. *significant difference at P < 0.05 by t-test.

The photosynthetic indexes of Tub and CK were measured (**Supplementary Table S5**). The results, under a light intensity of 1200µmol m^⁻²^ s^⁻¹^ and 800µmol mol^⁻¹^ CO_2_, show a significant increase in Gs, Ci, and Tr for Tub (**Figures 3A-C**), with respective increments of 60.74%, 37.70%, and 60.54%. However, the net photosynthetic rate (Pn) of Tub decreased compared to CK (**Figure 3D**). Further measurements on the dorsal and abdominal sides of Tub and CK revealed a significant decrease in abdominal Pn in Tub by 39.62%, while dorsal Pn showed no significant change. Notably, the dorsal PN of Tub was higher than its abdominal Pn, while CK exhibited higher abdominal Pn than dorsal Pn. Interestingly, the dorsal Pn of CK remained higher than the dorsal Pn of Tub (**Figure 3D**; **Supplementary Table S5**). In summary, these data suggest that the photosynthetic performance of Tub was relatively lower, especially on the internally curled abdominal side. However, Gs, Ci, and Tr of Tub were higher, which may be related to its unique leaf structure and stomatal distribution.

### 3.4 Analysis of endogenous hormone levels in *G. biloba* leaves with different leaf shapes

In the analysis of three groups of leaf samples, the content of eight common hormones, including auxin and GA, was determined. The data indicate that in normal fan-shaped leaves (CK6), the levels of ETH and GA were higher than those in two types of trumpet-shaped leaves (Tub19, Tub6). JA content was higher in Tub6 and Tub19 than in CK6. It is also noteworthy that auxin had the highest content among the measured hormones, with the highest concentration in Tub6 and the lowest in Tub19. ABA content was also highest in Tub6 and lowest in Tub19. The differences in SL content measured in the three samples were consistent with the ABA situation. The CKs showed the highest content in Tub19 and the lowest in Tub6 (**Table 1**).

**Table 1:** Endogenous hormone content data between different samples.

Class	Auxin(ng/g)	CKs (ng/g)	ETH(ng/g)	GA(ng/g)	JA(ng/g)	SL(ng/g)	ABA(ng/g)
Tub19	2477.56 ± 6.58	339.70 ± 0.50	113.75 ± 2.06	31.74 ± 1.29	373.94 ± 4.15	79.81 ± 1.59	426.10 ± 17.33
Tub6	5116.30 ± 15.76	99.20 ± 0.18	104.66 ± 2.85	30.82 ± 0.98	441.85 ± 3.24	126.51 ± 5.30	502.79 ± 24.24
CK6	2708.78 ± 2.31	102.80 ± 0.20	123.87 ± 6.16	35.30 ± 0.55	289.91 ± 4.98	88.43 ± 9.04	434.58 ± 13.22

In the analysis of hormone-like metabolites in various samples, targeted metabolic profiling was conducted, and differential hormones were screened using fold change values. In the Tub6vsCK6 comparison, 15 differential hormones were identified, with 4 upregulated and 11 downregulated; Tub19vsCK6 revealed 31 differential hormones, with 8 upregulated and 23 downregulated; Tub19vsTub6 showed 26 differential hormones, with 10 upregulated and 16 downregulated (**Supplementary Table S6**).

**Figure 4:**
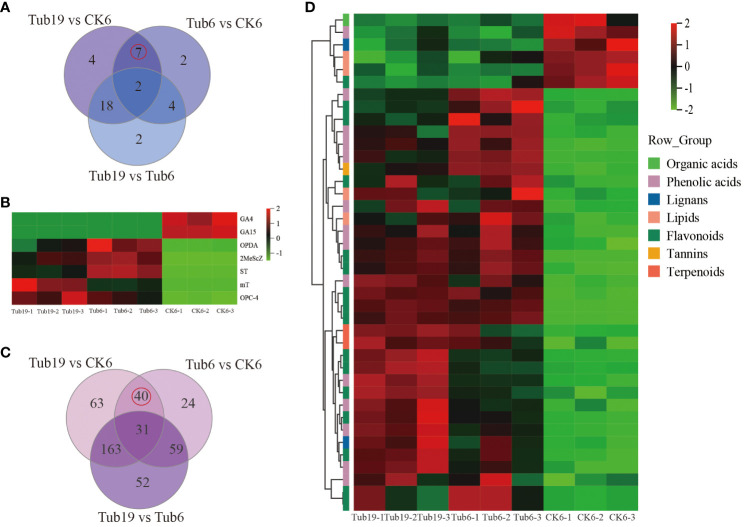
Screening and analysis of differential metabolites. **(A)** The Wayne diagram of differential hormones for each contrast combination. **(B)** Selected differential hormone heat map. **(C)** Wayne diagram of differential metabolites in each comparison combination. **(D)** Differential metabolite heat map. The horizontal represents the sample name, the vertical represents metabolite information, and different colors represent different values obtained after standardization of relative content (red represents high content, green represents low content).

The Venn diagram of three comparison groups (**Figure 4A**) identified 7 common differential hormones in the Tub19 vs CK6 and Tub6 vs CK6 combinations. Two CKs, mT and MeScZ, were detectable in Tub19 and Tub6 but had undetectable levels in normal fan-shaped leaves CK6; a similar pattern was observed for ST. Conversely, two GAs (GA4, GA15) showed undetectable levels in Tub19 and Tub6 but were detectable in CK6 with lower levels. Two JAs (OPC-4, OPDA) exhibited significantly higher levels in Tub19 and Tub6 compared to CK6, although CK6 had non-zero levels of these substances (**Figure 4B**; **Supplementary Table S7**).

### 3.5 Metabolomic analysis of *G. biloba* leaves with different leaf shapes

In this study, a total of 1302 metabolites were detected. In the Tub19 vs CK6 combination, 297 differential metabolites (DAMs) were identified, with 149 upregulated and 148 downregulated; Tub6 vs CK6 revealed 154 DAMs, with 24 upregulated and 130 downregulated; Tub19 vs Tub6 identified 305 DAMs, including 106 upregulated and 199 downregulated (**Table 2**). Differential gene expression volcano plots were generated based on expression levels and significance (**Supplementary Figure S2**).

**Table 2:** Comparison of differential metabolites.

Group name	All sig diff	Upregulated	Downregulated
Tub19 vs CK6	297	148	149
Tub6 vs CK6	154	24	130
Tub19 vs Tub6	305	199	106

Through the Venn diagram of three comparative combinations (**Figure 4C**), 40 DAMs common to the Tub6 vs CK6 and Tub19 vs CK6 comparisons between trumpet-shaped and normal fan-shaped leaves were identified, including flavonoids (16), lignans and coumarins (2), phenolic acids (14), organic acids (1), tannins (1), terpenoids (2), and lipids (4) (**Supplementary Table S8**).

Analysis of the selected 40 DAMs revealed that, except for one type of phenolic acid (Acetovanillone), one type of flavonoid (Quercetin-3-O-(2’’-O-acetyl)glucuronide), one type of lignan and coumarin (Matairesinol-4,4’-di-O-glucoside), one type of organic acid (3-Methylmalic acid*), and two types of lipids (20-Carboxyarachidonic Acid, Hydroxyicosanoic Acid), they were higher in content in normal fan-shaped leaves (**Figure 4D**). A volcano plot of differential gene expression was constructed based on the expression levels and significance of differentially expressed genes.

### 3.6 Transcriptomic analysis of *G. biloba* leaves with different leaf shapes

In this study, we identified DEGs in various comparison groups. In the Tub19 vs CK6 comparison group, 3214 DEGs were identified, comprising 2251 upregulated DEGs and 963 downregulated DEGs. In the Tub6 vs CK6 comparison group, 2690 DEGs were discovered, consisting of 1059 upregulated DEGs and 1631 downregulated DEGs. The Tub19 vs Tub6 comparison group revealed 3666 DEGs, comprising 2716 upregulated DEGs and 950 downregulated DEGs (**Table 3**). A volcano plot of differential gene expression was constructed based on the expression levels and significance of differentially expressed genes (**Supplementary Figure S3**).

**Table 3:** Number of differentially expressed genes in each comparison group.

Combination Name	Number of upregulated genes	Number of downregulated genes	Sum of differential genes
Tub19 vs CK6	2251	963	3214
Tub6 vs CK6	1059	1631	2690
Tub19 vs Tub6	2716	950	3666

**Figure 5:**
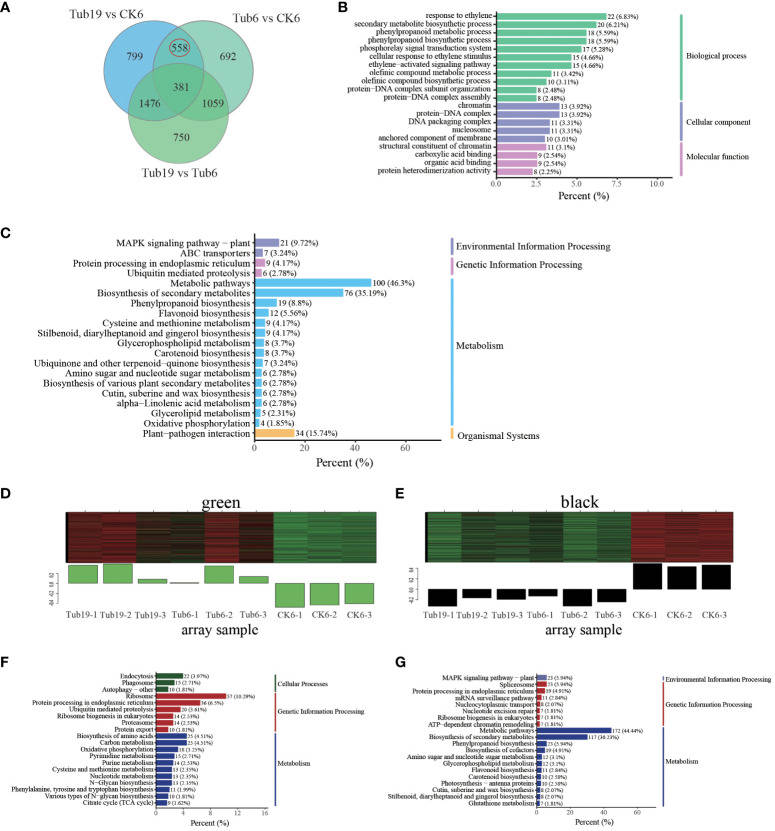
Screening and analysis of differential genes. **(A)** Venn diagram of differential genes for each comparison combination. **(B)** Differently expressed gene GO enrichment top20 bar chart. The horizontal coordinate indicates the number of differential genes annotated to the entry, and the vertical coordinate indicates the name of the GO entry. The numbers in the graph indicate the number of differentially expressed genes annotated to the entry, the ratio of the number of differentially expressed genes annotated to the GO entry to the total number of differentially expressed genes is shown in parentheses, and the label on the far right represents the classification to which the GO entry belongs. **(C)** Differential gene KEGG enrichment top20 bar chart. The horizontal coordinate represents the number of differential genes annotated to the pathway, and the vertical coordinate represents the name of the KEGG pathway. The numbers in the graph represent the number of differential genes annotated to the pathway, the ratio of the number of differential genes annotated to the pathway to the total number of differential genes is shown in the parentheses, and the label on the far right represents the classification to which the KEGG pathway belongs. **(D)** Specifically expressed gene module-green. The upper half of the image shows the clustering heatmap of genes within the module, with red indicating high expression and green indicating low expression. The lower half shows the expression patterns of module feature values in different samples. **(E)** Specifically expressed gene module-black. **(F)** TOP20 enrichment of green-specific gene modules KEGG. The horizontal axis represents the number of differentially expressed genes annotated to this pathway, and the vertical axis represents the name of the KEGG pathway. The numbers in the figure represent the number of differentially expressed genes annotated onto the pathway. The ratio of the number of differentially expressed genes annotated onto the pathway to the total number of differentially expressed genes is shown in parentheses. The rightmost label represents the classification of the KEGG pathway. **(G)** TOP20 enrichment of black specifically expressed gene module KEGG.

The primary focus of this research revolves around exploring the disparities between trumpet-shaped leaves (Tub19, Tub6) and the normal fan-shaped leaves (CK6). Differential gene expression analysis revealed the simultaneous presence of 558 DEGs in both Tub19 and Tub6 trumpet-shaped leaves, while these were absent in the normal CK6 fan-shaped leaves (**Figure 5A**). For a comprehensive comprehension of the biological processes related to G. *biloba* leaf curling, we performed GO enrichment analysis on the 558 DEGs. Particularly in the Biological Process category, significant enrichment was observed in processes such as response to ethylene (22, 6.83%), the phenylpropanoid biosynthetic process (18, 5.59%), and the secondary metabolite biosynthetic process (20, 6.21%). Significant enrichment in the Cellular Component category was found in the protein-DNA complex (13, 3.92%) and nucleosome (11, 3.31%). Meanwhile, the Molecular Function category highlighted the highest enrichment in genes associated with structural constituent of chromatin (11, 3.1%) (**Figure 5B**).

KEGG enrichment analysis was conducted on the selected 558 DEGs, and the top 20 significantly enriched pathway entries were displayed. The results indicate that the selected DEGs dominate various processes, including metabolic pathways (100; 46.3%), biosynthesis of secondary metabolites (76; 35.19%), plant-pathogen interaction (34; 15.74%), the MAPK signaling pathway in plants (21; 9.72%), phenylpropanoid biosynthesis (19; 8.8%), and flavonoid biosynthesis (12; 5.56%) (**Figure 5C**). In order to thoroughly investigate the factors influencing the formation of trumpet-shaped leaves in *G. biloba*, we conducted WGCNA module analysis on the selected DEGs, revealing 19 distinct modules. The green module signifies genes that exhibit high expression in trumpet-shaped leaves (Tub19, Tub6) and low expression in normal fan-shaped leaves (CK6) (**Figure 5D**). The black module indicates genes with low expression in trumpet-shaped leaves (Tub19, Tub6) and high expression in normal fan-shaped leaves (CK6) (**Figure 5E**). Subsequent KEGG enrichment analysis of genes in these two modules revealed that genes highly expressed only in trumpet-shaped leaves (Tub19, Tub6) were primarily involved in pathways such as the ribosome, protein processing in endoplasmic reticulum, biosynthesis of cofactors, and endocytosis (**Figure 5F**). Genes that are highly expressed in normal fan-shaped leaves (CK6) and lowly expressed in trumpet-shaped leaves (Tub19, Tub6) were primarily enriched in pathways related to hormone and metabolite synthesis, including phenylpropanoid biosynthesis, spliceosome, plant hormone signal transduction, and flavonoid biosynthesis (**Figure 5G**). This indicates that the curling of *G. biloba* leaves may be associated with material transport within the leaves and the content of secondary metabolites at different locations.

### 3.7 Combined analysis of the causes of the formation of Trumpet-shaped *G. biloba* leaves

#### 3.7.1 Analysis of differential genes, metabolites, and hormones involved in the formation of trumpet-shaped *G. biloba* leaves

We conducted KEGG enrichment analysis on differentially expressed genes and hormones from trumpet-shaped and normal fan-shaped leaves, all of which significantly enriched four pathways, encompassing biosynthesis of secondary metabolites, metabolic pathways, plant hormone signal transduction, and carotenoid biosynthesis (**Supplementary Figure S4A**). KEGG enrichment analysis of the measured DAMs and DEGs was performed and a total of 8 pathways were enriched, namely biosynthesis of secondary metabolites, metabolic pathways, flavonoid biosynthesis, 2-oxocarboxylic acid metabolism, and biosynthesis of amino acids, valine, leucine and isoleucine biosynthesis, cutin, suberine and wax biosynthesis, and tyrosine metabolism (**Supplementary Figure S2B**).The pathways enriched in differential hormones, DAMs, and DEGs include metabolic pathways (1 hormone, 4 metabolites, 100 genes) and secondary metabolite biosynthesis pathways (2 hormones, 4 metabolites, 76 genes). This suggests that the trumpet shape of *G. biloba* leaves is a result of variations in the content of certain secondary metabolites in the leaves, primarily involving plant hormones such as GA, auxin, and secondary metabolites such as flavonoids.

**Figure 6:**
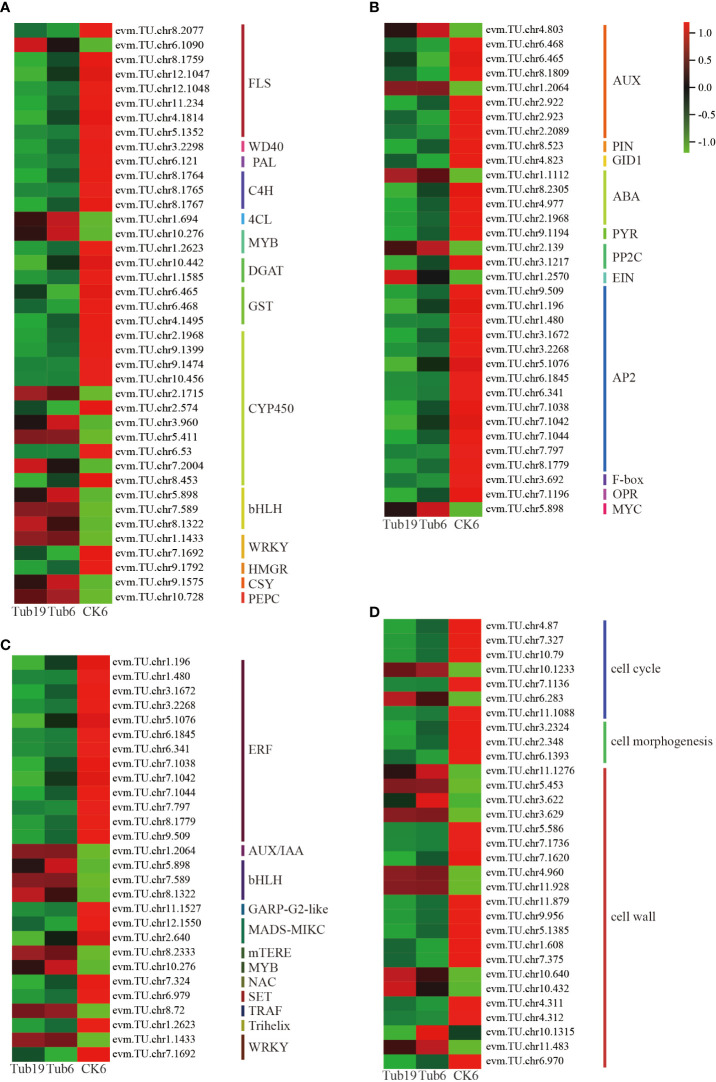
Analysis of differentially expressed genes associated with leaf curling in *G. biloba.* **(A)** Differential gene heat map with differential metabolites. **(B)** Differential gene heat map related to differential hormones. **(C)** Differential gene heat map of transcription factors. **(D)** Cell-related differential gene heat map.

We conducted an in-depth exploration of differentially expressed genes influencing differential metabolites such as flavonoids and phenolic acids (**Figure 6A**). Notably, there was a lower expression of genes including *FLS, WD40, PAL, C4H*, and *GST* in Tub, whereas *bHLH, CSY*, and *PEPC* were more highly expressed. Furthermore, genes such as *CYP450, MYB*, and *WRKY* exhibited mixed expression patterns, with some showing higher expression in Tub and others in CK.

Further analysis was conducted on genes influencing the levels of auxin, GA, and other hormones (**Figure 6B**). The findings revealed that genes associated with auxin synthesis predominantly exhibited heightened expression levels in CK. However, two specific genes, *evm.TU.chr4.803* and *evm.TU.chr1.2064*, demonstrated elevated expression in Tub. The receptor protein *GID1* of GA displayed higher expression in CK. Furthermore, genes related to ABA synthesis generally showed lower expression levels in Tub compared to CK. Examining ETH synthesis-related genes, the transcription factor *AP2* exhibited reduced expression in Tub, while *EIN* displayed heightened expression in Tub. Genes involved in JA synthesis, such as *OPDA*, exhibited heightened expression in CK, whereas *MYC* demonstrated elevated expression in Tub.

Transcription factors play a crucial role in various life activities of plants, exerting important functions in the growth and development processes of plants. The differentially expressed genes selected in this experiment include 13 classes of transcription factors, with 28 genes (**Figure 6C**). Analysis of these genes reveals that transcription factors such as *ERF, GARP-G2-like, MADS-MIKC, NAC*, and *SET* were lowly expressed in Tub, while *AUX/IAA, bHLH, mTERF, MYB*, and *TRAF* were highly expressed in Tub. *WRKY* transcription factors related to plant signal transduction showed one gene highly expressed in Tub and another with a higher expression in CK.

Finally, in order to gain a deeper understanding of the formation of trumpet-shaped G. *biloba* leaves, we investigated genes that affect the number of leaf cells, cell morphology, and plant cell wall synthesis (**Figure 6D**). The results show that cell cycle-related genes were expressed at higher levels in CK than in Tub. Only two genes regulating cell apoptosis, *evm.TU.chr10.1233* and *evm.TU.chr6.283*, were highly expressed in Tub. Genes related to cell morphology were also more highly expressed in CK than in Tub. Genes related to cell wall synthesis were mostly highly expressed in CK, including genes influencing the biological occurrence of cell walls, large-molecule synthesis of cell walls, and metabolic processes. Some genes related to cell wall modification were highly expressed in Tub.

#### 3.7.2 Key regulatory pathways of leaf morphology formation in *G. biloba*

**Figure 7:**
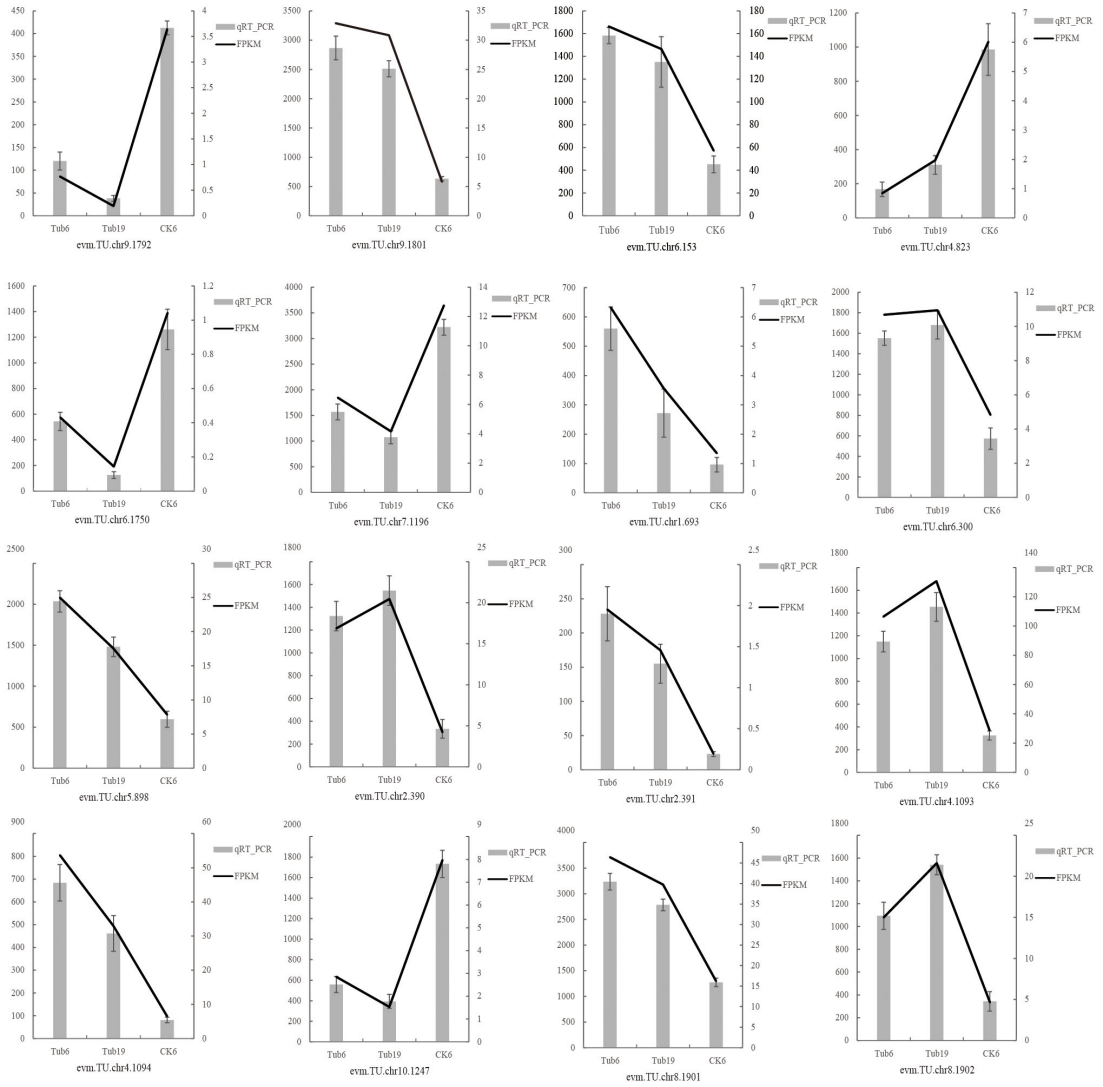
The key regulatory pathway of biosynthesis in the morphological formation of *G. biloba* leaves. Log_2_ (chromatographic peak area) represents the relative content of metabolites, Log_2_ (FPKM) represents gene expression, Tub19, Tub6, and CK6 represent different *G. biloba* leaf samples, the heat map represents the expression of each gene in the three samples, and the histogram represents the content of each hormone and metabolite in the three samples.

By synthesizing transcriptomic and metabolomic data, we established a regulatory network involving genes and metabolites in the formation of trumpet-shaped *G. biloba* leaves (**Figure 7**). The results reveal that cells in *G. biloba* leaves produce primary metabolites, including PEP, acetyl-CoA, and shikimate, through pathways such as glycolysis. Notably, acetyl-CoA and shikimate act as precursors in secondary metabolic pathways, influencing the expression of genes such as *KASI* and *AtMDD2*, as well as *HMDH*. Consequently, this regulatory network modulates the levels of plant hormones such as auxin and GA, as well as secondary metabolites such as flavonoids and phenolic acids. Within secondary metabolic pathways, *PAL* exhibited downregulation in Tub, while *4CL* and *C4H* showed upregulation. The differential expression of these genes impacts the levels of metabolites, including flavonoids, phenolic acids, and lignin in the downstream pathway of phenylpropane metabolism. As an illustration, the upregulation of *TAT* in Tub led to a higher content of the phenolic acid, salidroside, compared to CK. Similarly, the downregulation of genes such as *CYP73A, HST*, and *HCT* in Tub also resulted in higher levels of flavonoids, specifically sakuranetin and eriodictyol, compared to CK.

The experiment specifically focused on pathways related to plant hormone synthesis, including plant hormone signal transduction, tryptophan metabolism, diterpenoid biosynthesis, and carotenoid biosynthesis. The results indicate the early response gene *AUX* of auxin was upregulated in Tub, while the rapid response gene *SAUR*, which regulates cell growth, was downregulated. This ultimately led to higher levels of auxin (tryptophan, indoleacetic acid) in Tub compared to CK, and lower levels of tryptamine (auxin) in Tub than in CK. Additionally, in the plant hormone regulatory net-work, BR receptor protein *BRI1*, GA receptor protein *GID1*, genes related to ABA synthesis (*AtMO2, SILD*), and JA synthesis gene *OPDA* were all downregulated in Tub. Conversely, genes influencing JA (*HICCL5*), ABA receptor proteins (*PYR/PYL*), JA transcription factor *MYC2, ACC oxidase* involved in ETH synthesis, and *AtSDR2a* involved in ABA synthesis were upregulated in Tub.

These varying gene expression levels ultimately impacted the levels of ST and JA in Tub compared to CK, while GA15, GA4, 5-Deoxystrigol, and ETH had much higher levels in CK than in Tub. The *BRI1* receptor protein for BR in *G. biloba* leaf cells discriminated BR, activating downstream signal pathways, thereby affecting cell elongation and division. Moreover, overexpression of the *SAUR* protein also markedly stimulates cell growth. Ultimately, the variations in intracellular plant hormones and secondary metabolite levels impact the size, quantity, and structure of cells within plant leaves.

### 3.8 qRT-PCR analysis of differential gene expression

**Figure 8:**
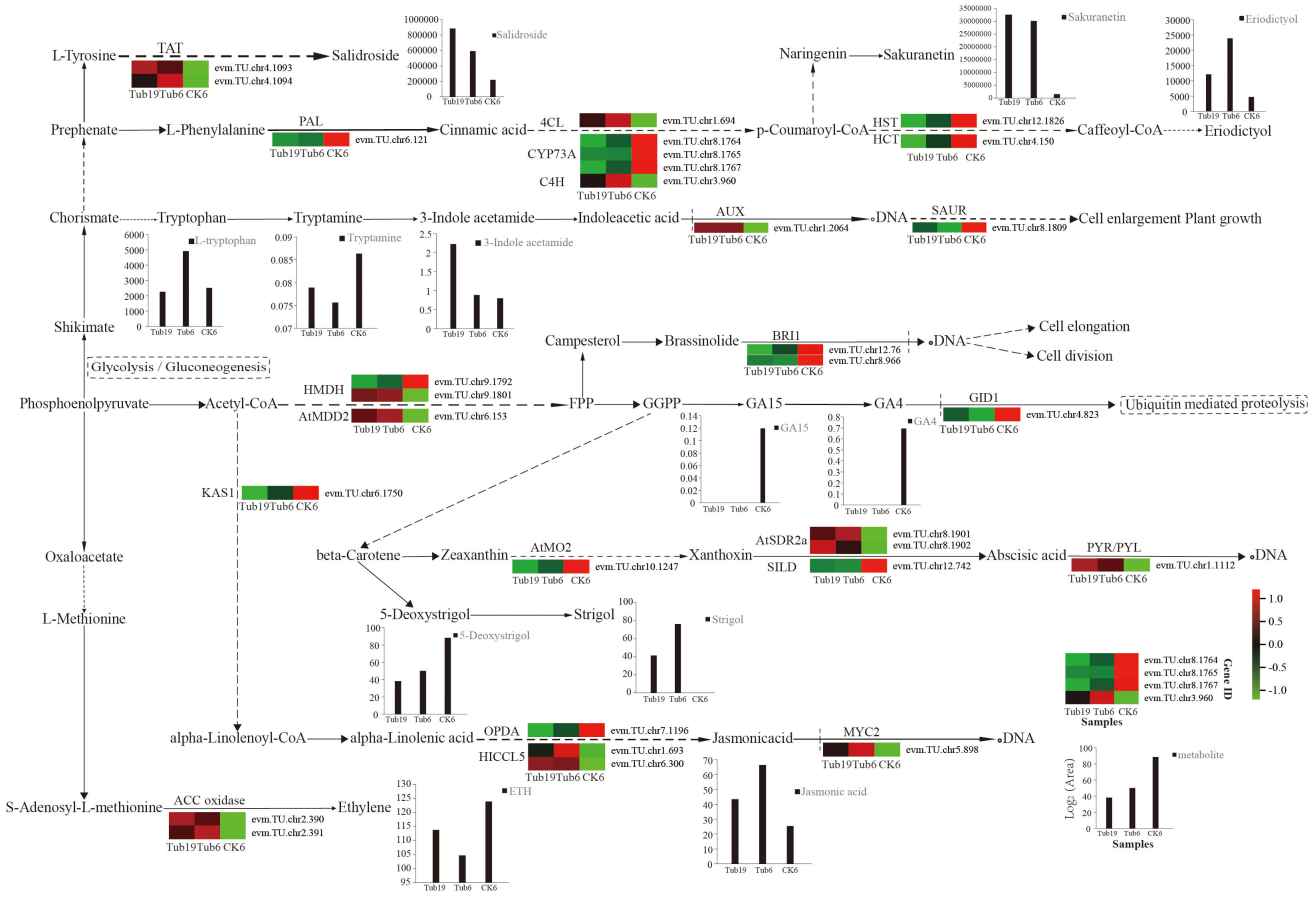
Verification of differentially expressed genes.

Sixteen differentially expressed genes were randomly selected for qRT-PCR validation, confirming the reliability of the transcriptome sequencing data. These genes include those involved in GA synthesis (*evm.TU.chr9.1792, evm.TU.chr9.1801, evm.TU.chr6.153, evm.TU.chr4.823*), JA synthesis (*evm.TU.chr6.1750, evm.TU.chr7.1196, evm.TU.chr1.693, evm.TU.chr6.300, evm.TU.chr5.898*), ethylene synthesis (*evm.TU.chr2.390, evm.TU.chr2.391*), salidroside synthesis (*evm.TU.chr4.1093, evm.TU.chr4.1094*), and ABA synthesis (*evm.TU.chr10.1247, evm.TU.chr8.1901, evm.TU.chr8.1902*). The validation results indicate that the expression trends of the 16 randomly selected genes in the samples are consistent with the RNA-seq expression levels (**Figure 8**).

## 4 Discussion

### 4.1 Trumpet-shaped leaf formation and phenotype

In comparison to normal fan-shaped leaves, *G. biloba* trumpet-shaped leaves exhibit a cylindrical curl, undergoing a morphological transition from a two-dimensional plane to a three-dimensional structure from CK to Tub. Anatomical studies indicate that changes in leaf morphology are associated with alterations in the number of epidermal cells in the leaf cross-section (Kuwabara et al., 2001). The alteration in leaf shapes in various plants is also linked to modifications in cell elongation (Kuwabara and Nagata, 2002). The anatomical experiments demonstrated significant differences in the size and thickness of epidermal cells on the dorsal and abdominal sides of Tub and CK leaves. Tub leaves exhibited smaller and fewer epidermal cells on the abdominal side.

The internal cellular structure of leaves not only mirrors the physiological functions of plants but also influences their photosynthetic capabilities (William W. and Terashima, 2018). Photosynthetic measurements indicated that the Pn of Tub leaves was lower compared to CK leaves, with a specific analysis showing a decrease of about 40% in the abdominal Pn of Tub leaves compared to CK. Observations of morphological and anatomical structures revealed that various indicators, such as leaf base angle, leaf weight, and leaf area, were smaller in Tub compared to CK. Anatomical experiments further showed that, in comparison to normal fan-shaped leaves, the volume of single-layer cells in the abdominal part of *G. biloba* trumpet-shaped leaves was increased, while the volume of single-layer cells in the dorsal part was decreased. This series of changes indicates that the increased cellular differences between the dorsal and abdominal parts of the leaves resulted in leaf curling, subsequently resulting in a reduction in the overall photosynthetic efficiency of the leaves. This reduction was accompanied by a decrease in nutrient supply within the leaves, ultimately leading to a reduction in the number of cells and a decrease in leaf volume in Tub.

### 4.2 The study of leaf morphology establishment

The development of leaves originates from the shoot apical meristem (SAM) during the post-embryonic development of plants, spanning four stages (Nakata et al., 2012). Asymmetric division and differentiation of leaf primordia lead to the formation of leaf organ plants (Jiajia et al., 2020). Current research on the proximal-distal axis pattern of plant leaves indicates three key pathways: TAS3–transacting small interfering R*NA–ARF3/ARF4, miR166–HD-ZIPIII*, and *AS1/AS2–KANADIs* (Kidner and Timmermans, 2007). The relevant genes involved in the proximal-distal axial pattern of plant leaves include *KANADI, AS1, AS2, ARF3, ARF4, TAS3, HD-ZIP III* family genes (*REV, PHB, PHV*); members of the YABBY family; and small RNAs, including *miR165/166, miR390, and ta-siRNA* (Singh et al., 2018; H. F. Wang et al., 2021). The proximal fate of the leaf depends on the activity of a few gene products, such as *PHV, PHB*, and *REV* from *HD-ZIP III* (McConnell et al., 2001; Emery et al., 2003). Furthermore, the distal fate of the leaf is controlled by genes such as *KAN, YABBY (YAB)*, and *ETT/ARF3-ARF4* (Kerstetter et al., 2001; Emery et al., 2003; Canales et al., 2005; Pekker et al., 2005). Abundant research indicates that leaf primordia establish proximal-distal polarity prior to initiating growth (Jiajia et al., 2020).

Recently, Liu H.L. and colleagues identified counterparts of three genes in the whole genome of *G. biloba*, namely *REV (evm.TU.chr10.207), ATHB8/ATHB15 (evm.TU.chr5.833, evm.TU.chr1.647)*, and *YABBY (evm.TU.chr7.1888, evm.TU.chr3.1783)*, which influence the leaf morphology of *G. biloba.* However, the key genes related to the proximal-distal axis pattern influencing *G. biloba* leaf formation have not yet been identified (Liu et al., 2021). In this experiment, no differentially expressed genes between trumpet-shaped leaves (Tub6, Tub19) and normal fan-shaped leaves (CK6) were found to be related to the establishment of proximal-distal polarity in leaves. Therefore, it can be reasonably inferred that the SAM had already determined the developmental morphology of *G. biloba* leaves in the early stages of leaf development. It is possible that the trumpet-shaped leaves of *G. biloba* may have established the proximal-distal pattern during the leaf primordium stage, thereby determining the final developmental morphology of the leaves as trumpet-shaped.

### 4.3 Molecular regulatory mechanisms for the establishment of trumpet-shaped leaf morphology

The formation and development of leaves in plants are intricately regulated by endogenous hormones (Santner and Estelle, 2009). Plant hormones such as ABA, ETH, and GA are closely related to the formation of heteromorphic leaves (Koenig and Sinha, 2010).Research has found that in the aquatic leaves of *Batrachium trichophyllum*, ETH affects the direction of leaf development by regulating the expression of morphogenesis genes (Juhyun et al., 2018). JA also exerts a significant influence on leaf development and cell metabolism by regulating the cell cycle (Mandaokar et al., 2010; Huang et al., 2017). In the trumpet-shaped leaves of *G. biloba*, JA levels surpass those in normal leaves, impacting the expression of cell cycle-related proteins and suppressing cell division (Pauwels et al., 2008). Endogenous hormones in plants can also cooperate to collectively regulate leaf development (Mazzoni-Putman et al., 2021). Auxin within plant cells stimulates the expression of *BRI1*, thereby reinforcing *BR* signal transduction (Zhang et al., 2021). The *BRI1-like* gene plays a critical role in balancing auxin and *BR* during the regulation of *G. biloba* leaf development (**Figure 7**).

The establishment of plant leaf morphology is significantly influenced by auxin (Arun Sampathkumar et al., 2014; Jan, 2017). Auxin plays an important role in the acidification of the cell wall (Fambrini and Pugliesi, 2013), the regulation of the microstructural domains of the cell wall (Dauphin et al., 2022), and the influence on the mechanical properties of the extracellular matrix and cell wall (François et al., 2023). However, the effects of auxin vary depending on developmental stages, tissue types, and organs (Majda and Robert, 2018). Auxin exhibits pleiotropic effects, promoting cell elongation in shoots (Lin et al., 2021) and induces cell wall acidification (Du et al., 2020), whereas in roots it induces cell wall alkalinization (Moreau et al., 2021; Tsai and Schmidt, 2021) and inhibits root cell expansion (Fendrych et al., 2018; L. X. Li et al., 2021). Studies on *Arabidopsis* revealed a higher susceptibility of the epidermis to auxin-induced cell wall gene regulation (Bargmann et al., 2013). Thus, within *G. biloba* trumpet-shaped leaves, the auxin content in the abdominal region of leaves might reinforce the rigidity of epidermal cell walls, while the auxin content in the dorsal region can acidify the epidermal cell walls, resulting in decreased intercellular interaction forces. Additionally, ion concentration also influences cell wall rigidity. High concentrations of Ca^2+^ can stimulate the demethylesterification process of PME, hardening the cell wall (X. Wang et al., 2020). The differential genes between trumpet-shaped and normal fan-shaped leaves involved a plethora of Ca^2+^ binding proteins. Variations in Ca^2+^ concentrations among leaf types could result in distinct mechanical properties of cell walls.

The establishment of plant leaf morphology is a complex process involving the coordinated regulation of multiple endogenous hormones and ion concentrations. Differences in endogenous hormone levels and ion concentrations, such as GA, BR, and auxin, between *G. biloba* trumpet-shaped and normal fan-shaped leaves may affect cell differentiation, proliferation, cell wall properties, and intercellular interaction forces, ultimately influencing the phenotypic characteristics of the leaves and giving rise to the characteristic trumpet-shaped leaf morphology.

## 5 Conclusions

The differential expression of genes such as *AUX/IAA, SAUR, GID1*, and *C4H* in trumpet-shaped leaves of *G. biloba* influences the levels of plant hormones such as auxin and GA, as well as flavonoids, phenolic acids, and other metabolites. This results in the elongation of the leaf cell division cycle and a reduction in cell number. Cell wall extension and cell expansion are also affected, leading to an increase in cell volume. Changes in auxin content, low expression of genes related to the cell cycle and cell wall synthesis, along with high expression of genes related to cell wall modification in the trumpet-shaped leaves of *G. biloba*, alter cell wall strength and intercellular interactions. This increases the morphological differences in epidermal cells between the dorsal and abdominal sides of the leaf, enhancing rigidity in the abdominal epidermal cell walls and increasing intercellular interaction forces. Meanwhile, the dorsal epidermal cell walls undergo acidification, leading to decreased intercellular interaction forces and ultimately causing leaf curling.

## Data availability statement

The datasets presented in this study can be found in online repositories. The names of the repository/repositories and accession number(s) can be found in the article/**Supplementary Material**.

## Author contributions

X-hL: Data curation, Formal analysis, Investigation, Software, Validation, Writing – original draft, Writing – review & editing. X-jK: Data curation, Investigation, Validation, Writing – review & editing. X-yZ: Data curation, Investigation, Software, Writing – review & editing. L-nS: Formal analysis, Investigation, Writing – review & editing. XB: Formal analysis, Software, Writing – review & editing. R-lW: Investigation, Software, Writing – review & editing. S-yX: Funding acquisition, Resources, Supervision, Writing – review & editing. L-mS: Conceptualization, Funding acquisition, Methodology, Project administration, Resources, Supervision, Visualization, Writing – review & editing.

## Supplementary material

Supplementary Table 1 Anthocyanin candidate gene primers.

Supplementary Table 2 qRT-PCR reaction system and reaction protocol.

Supplementary Table 3 Analysis of phenotypic characters of *G. biloba* leaves with different shapes. Note: * * indicates that the difference is extremely significant at the level of α = 0.05, * indicates that the difference is significant at the level of α = 0.05, the same below.

Supplementary Table 4 Anatomical data of different samples

Supplementary Table 5 Photosynthetic data of different leaf shapes

Supplementary Table 6 Comparison of differential hormones

Supplementary Table 7 The expression of 7 differential metabolites screened out

Supplementary Table 8 Statistical table of differential metabolites

## References

[B1] Arun Sampathkumar Yan. A. Krupinski P. Meyerowitz E. M. (2014). Physical forces regulate plant development and morphogenesis. Curr. Biol. 24, R475–R483. doi: 10.1016/j.cub.2014.03.014 24845680 PMC4049271

[B2] Bargmann B. O. R. Vanneste S. Krouk G. Nawy T. Efroni I. Shani E.. (2013). A map of cell type-specific auxin responses. Mol. Syst. Biol. 9, 688. doi: 10.1038/msb.2013.40 24022006 PMC3792342

[B3] Beerling D. J. Osborne C. P. Chaloner W. G. (2001). Evolution of leaf-form in land plants linked to atmospheric CO_2_ decline in the Late Palaeozoic era. Nature 410, 352–354. doi: 10.1038/35066546 11268207

[B4] Canales C. Grigg S. Tsiantis M. (2005). The formation and patterning of leaves: Recent advances. Planta 221, 752–756. doi: 10.1007/s00425-005-1549-x 15909148

[B5] Cao Z. Y. Su L. N. Zhang Q. Zhang X. Y. Kang X. J. Li X. H.. (2023). The development and transcriptome regulation of the secondary trunk of *Ginkgo biloba* L. Front. Plant Sci. 14. doi: 10.3389/fpls.2023.1161693 PMC10267747 37324703

[B6] Chen C. Shen Y. Yang W. Pan Q. Li C. Sun Q.. (2022). Comparative metabolic study of two contrasting chinese cabbage genotypes under mild and severe drought stress. Int. J. Mol. Sci. 23, 5947. doi: 10.3390/ijms23115947 35682623 PMC9180449

[B7] Chen W. Gong L. Guo Z. L. Wang W. S. Zhang H. Y. Liu X. Q.. (2013). A novel integrated method for large-scale detection, identification, and quantification of widely targeted metabolites: application in the study of rice metabolomics. Mol. Plant 6, 1769–1780. doi: 10.1093/mp/sst080 23702596

[B8] Dauphin B. G. Ranocha P. Dunand C. Burlat V. (2022). Cell-wall microdomain remodeling controls crucial developmental processes. Trends Plant Sci. 27, 1033–1048. doi: 10.1016/j.tplants.2022.05.010 35710764

[B9] Du M. Spalding E. P. Gray W. M. (2020). Rapid auxin-mediated cell expansion. Annu. Rev. Plant Biol. 71, 379–402. doi: 10.1146/annurev-arplant-073019-025907 PMC7733314 32131604

[B10] Emery J. F. Floyd S. K. Alvarez J. Eshed Y. Hawker N. P. Izhaki A.. (2003). Radial patterning of *Arabidopsis* shoots by class III HD-ZIP and KANADI genes. Curr. Biol. 13, 1768–1774. doi: 10.1016/j.cub.2003.09.035 14561401

[B11] Fambrini M. Pugliesi C. (2013). Usual and unusual development of the dicot leaf: involvement of transcription factors and hormones. Plant Cell Rep. 32, 899–922. doi: 10.1007/s00299-013-1426-1 23549933

[B12] Fang J. Guo T. Xie Z. Chun Y. Li X. (2021). The URL1–ROC5–TPL2 transcriptional repressor complex represses the *ACL1* gene to modulate leaf rolling in rice. Plant Physiol. 185, 1722–1744. doi: 10.1093/plphys/kiaa121 33793928 PMC8133684

[B13] Fendrych M. Akhmanova M. Merrin J. Glanc M. Hagihara S. Takahashi K.. (2018). Rapid and reversible root growth inhibition by TIR1 auxin signalling. Nat. Plants 4, 453–459. doi: 10.1038/s41477-018-0190-1 29942048 PMC6104345

[B14] François J. Yadav. S. Robert Stéphanie (2023). Auxin as an architect of the pectin matrix. J. Exp. Bot. 74, 6933–6949. doi: 10.1093/jxb/erad174 37166384 PMC10690733

[B15] He P. Zhang Y. Z. Li H. B. Fu X. Shang H. H. Zou C. S.. (2021). GhARF16-1 modulates leaf development by transcriptionally regulating the *GhKNOX2-1* gene in cotton. Plant Biotechnol. J. 19 (3), 548–562. doi: 10.1111/pbi.13484 32981232 PMC7955886

[B16] Hou J. Xu Y. Zhang S. Yang X. Wang S. Hong J.. (2023). Auxin participates in regulating the leaf curl development of Wucai (*Brassica campestris L.*). Physiologia Plantarum 175 (2), e13908. doi: 10.1111/ppl.13908 37022777

[B17] Huang H. Liu B. Liu L. Y. Song S. S. (2017). Jasmonate action in plant growth and developmen. J. Exp. Bot. 68, 1349–1359. doi: 10.1093/jxb/erw495 28158849

[B18] Jan T. (2017). Plant development: from dynamics to mechanics. Curr. Biol. 27, R313–R315. doi: 10.1016/j.cub.2017.02.062 28441567

[B19] Jiajia W. Jing X. A. Qian Q. Guang-heng Z. (2020). Development of rice leaves: how histocytes modulate leaf polarity establishment. Rice Sci. 27, 468–479. doi: 10.1016/j.rsci.2020.09.004

[B20] Kanehisa M. S. (2000). KEGG: kyoto encyclopedia of genes and genomes. Nucleic Acids Res. 28, 27–30. doi: 10.1093/nar/28.1.27 10592173 PMC102409

[B21] Kerstetter R. A. Bollman K. Taylor A. R. Bomblies K. Poethig R. S. (2001). *KANADI* regulates organ polarity in *Arabidopsis* . Nature 411, 706–709. doi: 10.1038/35079629 11395775

[B22] Kidner C. A. Timmermans M. C. (2007). Mixing and matching pathways in leaf polarity. Curr. Opin. Plant Biol. 10, 13–20. doi: 10.1016/j.pbi.2006.11.013 17140842

[B23] Kim D. Landmead B. Salzberg S. L. (2015). HISAT: a fast spliced aligner with low memory requirements. Nat. Methods 12, 357–360. doi: 10.1038/nmeth.3317 25751142 PMC4655817

[B24] Kim J. Joo Y. Kyung J. Jeon M. Park J. Y. Lee H. G.. (2018). A molecular basis behind heterophylly in an amphibious plant, *Ranunculus trichophyllus* . PloS Genet. 14 (2). doi: 10.1371/journal.pgen.1007208 PMC5831646 29447166

[B25] Koenig D. Sinha N. (2010). Evolution of leaf shape: A pattern emerges. Curr. Topics Dev. Biol. 91, 169–183. doi: 10.1016/S0070-2153(10)91006-5 20705182

[B26] Kuwabara A. Nagata T. (2002). Views on developmental plasticity of plants through heterophylly. Recent Res. Dev. Plant Physiol. 3, 45–59.

[B27] Kuwabara A. Tsukaya H. Nagata T. (2001). Identification of factors that cause heterophylly in *ludwigia arcuata* walt. (Onagraceae). Plant Biol. 3, 98–105. doi: 10.1055/s-2001-11748

[B28] Nikolov L. A. Runions A Das Gupta M. Tsiantis M. (2019). Chapter Five - Leaf development and evolution. Current Topics in Developmental Biology 131, 109–139. doi: 10.1016/bs.ctdb.2018.11.006 30612614

[B29] Li L. X. Verstraeten I. Roosjen M. Takahashi K. Rodriguez L. Merrin J.. (2021). Cell surface and intracellular auxin signalling for H^+^ fluxes in root growth. Nature 599 (7884), 273–277. doi: 10.1038/s41586-021-04037-6 34707283 PMC7612300

[B30] Li Y. Zhou C. X. Yan X. J. Zhang J. R. Xu J. L. (2016). Simultaneous analysis of ten phytohormones in Sargassum horneri by high-performance liquid chromatography with electrospray ionization tandem mass spectrometry. J. Separation Sci. 39, 1804–1813. doi: 10.1002/jssc.201501239 26990813

[B31] Lin W. Zhou X. Tang W. Takahashi K. Pan X. Dai J.. (2021). TMK-based cell-surface auxin signalling activates cell-wall acidification. Nature 599, 278–282. doi: 10.1038/s41586-021-03976-4 34707287 PMC8549421

[B32] Liu H. Wang X. Wang G. Cui P. Cao F. (2021). The nearly complete genome of *Ginkgo biloba* illuminates gymnosperm evolution. Nat. Plants 7, 748–756. doi: 10.1038/s41477-021-00933-x 34135482

[B33] Love M. I. Huber W. Anders S. (2014). Moderated estimation of fold change and dispersion for RNA-seq data with DESeq2. Genome Biol. 15, 550. doi: 10.1186/s13059-014-0550-8 25516281 PMC4302049

[B34] Majda M. Robert S. (2018). The role of auxin in cell wall expansion. Int. J. Mol. Sci. 19, 951. doi: 10.3390/ijms19040951 29565829 PMC5979272

[B35] Mandaokar A. Thines B. Shin B. Lange B. M. Choi G. Koo Y. J.. (2010). Transcriptional regulators of stamen development in *Arabidopsis* identified by transcriptional profiling. Plant J. Cell Mol. Biol. 46, 984–1008. doi: 10.1111/j.1365-313X.2006.02756.x 16805732

[B36] Mazzoni-Putman S. M. Brumos J. Zhao C. S. Alonso J. M. Stepanova A. N. (2021). Auxin interactions with other hormones in plant development. Cold Spring Harbor Perspect. Biol. 13 (10), a039990. doi: 10.1101/cshperspect.a039990 PMC8485746 33903155

[B37] McConnell J. R. Emery J. Eshed Y. Bao N. Bowman J. Barton M. K.. (2001). Role of *PHABULOSA* and *PHAVOLUTA* in determining radial patterning in shoots. Nature 411, 709–713. doi: 10.1038/35079635 11395776

[B38] Moreau H. Zimmermann S. D. Gaillard I. Paris N. (2021). pH biosensing in the plant apoplast - a focus on root cell elongation. Plant Physiol. 187, 504–514. doi: 10.1093/plphys/kiab313 35237817 PMC8491080

[B39] Nakata M. Matsumoto N. Tsugeki R. Rikirsch E. Okada K. (2012). Roles of the middle domain-specific *WUSCHEL-RELATED HOMEOBOX* genes in early development of leaves in *Arabidopsis* . Plant Cell 24 (4), 519–535. doi: 10.1105/tpc.111.092858 22374393 PMC3315230

[B40] Pauwels L. Morreel K. De Witte E. Lammertyn F. Van Montagu M. Boerjan W.. (2008). Mapping methyl jasmonate-mediated transcriptional reprogramming of metabolism and cell cycle progression in cultured *Arabidopsis* cells. Proc. Natl. Acad. Sci. U. S. A. 105 (4), 1380–1385. doi: 10.1073/pnas.0711203105 18216250 PMC2234147

[B41] Pekker I. Alvarez J. P. Eshed Y. (2005). Auxin response factors mediate *arabidopsis* organ asymmetry via modulation of KANADI activity. Plant Cell Online 17, 2899–2910. doi: 10.1105/tpc.105.034876 PMC1276018 16199616

[B42] Radwan D. E. M. Fayez K. A. (2016). Photosynthesis, antioxidant status and gas-exchange are altered by glyphosate application in peanut leaves. Photosynthetica 54, 307–316. doi: 10.1007/s11099-016-0075-3

[B43] Santner A. Estelle M. (2009). Recent advances and emerging trends in plant hormone signalling. Nature 459, 1071–1078. doi: 10.1038/nature08122 19553990

[B44] Simura J. Antoniadi I. Siroká J. Tarkowská D. Strnad M. Ljung K.. (2018). Plant hormonomics: multiple phytohormone profiling by targeted metabolomics. Plant Physiol. 177, 476–489. doi: 10.1104/pp.18.00293 29703867 PMC6001343

[B45] Singh A. Gautam V. Singh S. Sarkar Das S. Verma S. Mishra V.. (2018). Plant small RNAs: advancement in the understanding of biogenesis and role in plant development. Planta: Int. J. Plant Biol. 248, 545–558. doi: 10.1007/s00425-018-2927-5 29968061

[B46] Tang F. Sun P. Zhang Q. Zhong F. Wang Y. Lu M.. (2022). Insight into the formation of trumpet and needle-type leaf in *Ginkgo biloba* L. mutant. Front. Plant Sci. 13, 1081280. doi: 10.3389/fpls.2022.1081280 36570947 PMC9780455

[B47] Tsai H. H. Schmidt W. (2021). The enigma of environmental pH sensing in plants. Nat. Plants 7, 106–115. doi: 10.1038/s41477-020-00831-8 33558755

[B48] Tsukaya H. (2018). Leaf shape diversity with an emphasis on leaf contour variation, developmental background, and adaptation. Semin. Cell Dev. Biol. 79, 48–57. doi: 10.1016/j.semcdb.2017.11.035 29199138

[B49] Varet H. Brillet-Guéguen L. Coppée J. Y. Dillies M. A. (2016). SARTools: A DESeq2-and edgeR-based R pipeline for comprehensive differential analysis of RNA-seq data. PloS One 11 (6), e0157022. doi: 10.1371/journal.pone.0157022 27280887 PMC4900645

[B50] Wang H. F. Kong F. J. Zhou C. E. (2021). From genes to networks: The genetic control of leaf development. J. Integr. Plant Biol. 63, 1181–1196. doi: 10.1111/jipb.13084 33615731

[B51] Wang X. Wilson L. Cosgrove D. J. (2020). Pectin methylesterase selectively softens the onion epidermal wall yet reduces acid-induced creep. J. Exp. Bot. 71, 2629–2640. doi: 10.1093/jxb/eraa059 32006044 PMC7210771

[B52] William W. Adams I. T. III (2018). The Leaf: A Platform for Performing Photosynthesis. Cham, Switzerland: Springer. 44, 575. doi: 10.1007/978-3-319-93594-2

[B53] Xiao H. M. Cai W. J. Ye T. T. Ding J. Feng Y. Q. (2018). Spatio-temporal profiling of abscisic acid, indoleacetic acid and jasmonic acid in single rice seed during seed germination. Analytica Chimica Acta 1031, 119–127. doi: 10.1016/j.aca.2018.05.055 30119729

[B54] Xingwen Z. (2006). Development and Evolution of Leaves of Ginkgo Biloba. J. Shenyang Univ. 18 (4), 83–86. Available at: https://api.semanticscholar.org/CorpusID:87611798.

[B55] Xu Y. Kong W. Wang F. Wang J. Tao Y. Li W.. (2021). Heterodimer formed by ROC8 and ROC5 modulates leaf rolling in rice. Plant Biotechnol. J. 19, 2662–2672. doi: 10.1111/pbi.13690 34448351 PMC8633501

[B56] Yuan Y. Ren S. Liu X. Su L. Wu Y. Zhang W.. (2022). SlWRKY35 positively regulates carotenoid biosynthesis by activating the MEP pathway in tomato fruit. New Phytol. 234, 164–178. doi: 10.1111/nph.17977 35048386

[B57] Zhang J. Zhang Y. Khan R. Wu X. Zhou L. Xu N.. (2021). Exogenous application of brassinosteroids regulates tobacco leaf size and expansion via modulation of endogenous hormones content and gene expression. Physiol. Mol. Biol. Plants 27, 847–860. doi: 10.1007/s12298-021-00971-x 33967467 PMC8055801

[B58] Zhiyan Z. Zheng S. (2003). The missing link in *Gingo* evolution. Nature 423, 821–822. doi: 10.1038/423821a 12815417

[B59] Zhou J. Liu C. Chen Q. Liu L. Niu S. Chen R.. (2022). Integration of rhythmic metabolome and transcriptome provides insights into the transmission of rhythmic fluctuations and temporal diversity of metabolism in rice. Sci. China-Life Sci. 65, 1794–1810. doi: 10.1007/s11427-021-2064-7 35287184

