# Supplementary material for: Formation mechanism and regulation analysis of trumpet leaf in Ginkgo biloba L
Source: Front Plant Sci. 2024 Jul 17;15:1367121. doi: 10.3389/fpls.2024.1367121 (PMC11288918; doi:10.3389/fpls.2024.1367121)
Supplement: Supplementary Table 1 — Anthocyanin candidate gene primers. [file Table_1.pdf]

Table S1. Anthocyanin candidate gene primers.

Gene ID	Forward primers（5’-3’）	Reverse primers（5’-3’)
evm.model.chr9.1792	GGGGAGTAATGCAGGAGAGT	AGGAACACTTTGTTCATCAA
evm.model.chr9.1801	TGCATGTGGTGAGCCTGGGA	TCAACTCCTCCCTTCTTTCA
evm.model.chr6.153	CGCAGCCTCTATGGTGGATT	ACGTGAGCTTACCACAGCAA
evm.model.chr4.823	ATCACATTGGGTGCAGTCGT	CTCGAATCTCCAGGGGCTTG
evm.model.chr6.1750	GTCATGGGTGAGGGTTCTGG	TGCGATTATCGGTGCTCCTC
evm.model.chr7.1196	TTCGCACTGCTGCAAGAAAC	CTTCCACTACCTCAAGCGCA
evm.model.chr1.693	TGGCAGTTGCAGCTGGATTA	TGCAGCGCTGATAATTGCAC
evm.model.chr6.300	TGGCCAAATTGCACTCGTTG	TTCTGAACTCCAAGGTCGGC
evm.model.chr5.898	GCCCTCCGAAGGTCTAACAG	AGAGTTTGGGAAGTGGGCAG
evm.model.chr2.390	GGTCTACGTGCACACACTGA	TACCGTTGCTAATCGCCTCC
evm.model.chr2.391	ATCAGCTGGAGGCGATTAGC	CACTGCATCGACTGATGGGT
evm.model.chr4.1093	ATTGCTGTGCATAACCGGGA	CATTCGCTCCTTGAACCCCT
evm.model.chr4.1094	ATGCACACATGGTGTTTGGC	GCAAGCAACAAGCCATCCAA
evm.model.chr10.1247	GTTCAACAAGGTTCGTGGGC	CACAGTCCACTTGTGTGGGA
evm.model.chr8.1901	AGAGGTGGTTAATGGCGGTG	TGCGATGTCCGCGATAATGA
evm.model.chr8.1902	TGGTGCAGAGGTGGCTAATG	TGCGATGTCCGCGATAATGA
