# Supplementary material for: Formation mechanism and regulation analysis of trumpet leaf in Ginkgo biloba L
Source: Front Plant Sci. 2024 Jul 17;15:1367121. doi: 10.3389/fpls.2024.1367121 (PMC11288918; doi:10.3389/fpls.2024.1367121)
Supplement: Supplementary Table 2 — qRT-PCR reaction system and reaction protocol. [file Table_2.pdf]

Table S2. qRT-PCR reaction system and reaction protocol.

The reaction system		The reaction procedure		
Component	Volume	Temperature	Duration	Number of cycles
cDNA template	1 μL	94℃	30 sec	1
Upstream primer（10 μM）	0.4 μL			
Downstream primer（10 μM）	0.4 μL	94℃	5 sec	40-45
2×PerfectStart Green qPCR SuperMix	10 μL			
ddH2O	8.2 μL	60℃	30 sec	40-45
Total	20 μL			
