# Supplementary material for: Formation mechanism and regulation analysis of trumpet leaf in Ginkgo biloba L
Source: Front Plant Sci. 2024 Jul 17;15:1367121. doi: 10.3389/fpls.2024.1367121 (PMC11288918; doi:10.3389/fpls.2024.1367121)
Supplement: Supplementary Table 3 — Analysis of phenotypic characters of G. biloba leaves with different shapes. Note: * * indicates that the difference is extremely significant at the level of α = 0.05, * indicates that the difference is significant at the level of α = 0.05, the same below. [file Table_3.pdf]

Table S3 Analysis of phenotypic characters of *G. biloba* leaves with different shapes

Group	Leaf base angle (°)	Leaf area (mm^2)	Leaf fresh weight (g)	Leaf thickness(mm)
Tub19	42.67±9.46	69119.83±11788.35	0.18±0.03	0.29±0.00
Tub 6	14.03±3.44	56767.33±22131.21	0.21±0.06	0.32±0.01
CK6	318.87±20.27**	225631.33±27947.74**	0.66±0.03**	0.27±0.00

Note: * * indicates that the difference is extremely significant at the level of α = 0.05,
