# Supplementary material for: Formation mechanism and regulation analysis of trumpet leaf in Ginkgo biloba L
Source: Front Plant Sci. 2024 Jul 17;15:1367121. doi: 10.3389/fpls.2024.1367121 (PMC11288918; doi:10.3389/fpls.2024.1367121)
Supplement: Supplementary Table 4 — Anatomical data of different samples [file Table_4.pdf]

Table S4 Anatomical data of different samples

**sample**	**Tub19**	**Tub6**	**CK6**
Single layer cell thickness of abdominal epidermis（μm）	30.198±0.314a	28.980±1.4 87a	21.980±0.329b
Single layer cell thickness of dorsal epidermis（μm）	15.369±0.922b	13.485±1.422b	20.722±1.806a
Single cell area of abdominal epidermis（μm^2）	50.650±4.946a	45.667±10.092a	37.812±0.421a
Single cell area of dorsal epidermis（μm^2）	20.701±3.691ab	23.499±1.155a	25.009±0.649b
