# Supplementary material for: Formation mechanism and regulation analysis of trumpet leaf in Ginkgo biloba L
Source: Front Plant Sci. 2024 Jul 17;15:1367121. doi: 10.3389/fpls.2024.1367121 (PMC11288918; doi:10.3389/fpls.2024.1367121)
Supplement: Supplementary Table 5 — Photosynthetic data of different leaf shapes [file Table_5.pdf]

Table S5 Photosynthetic data of different leaf shapes

**photosynthetic index**		**Tub**	**CK**
transpiration rate (Tr) (m mol m^{-2} s^{-1})		0.891±0.076*	0.555±0.0076
the intercellular CO_2 concentration (Ci)(μmol mol^{-1})		1099.661±66.125*	798.611±58.170
stomata conductance (Gs) (mol m^{-2} s^{-1})		0.030±0.003*	0.019±0.000
net photosynthetic rate (Pn) (mol m^{-2} s^{-1})	abdominal /Upper epidermis	5.976±0.960	9.897±1.047*
	dorsal /lower epidermis	6.488±1.598	7.455±1.762

Note: * indicates that the difference is significant at the level of $\alpha = 0.05$, the same below.
