# Supplementary material for: Formation mechanism and regulation analysis of trumpet leaf in Ginkgo biloba L
Source: Front Plant Sci. 2024 Jul 17;15:1367121. doi: 10.3389/fpls.2024.1367121 (PMC11288918; doi:10.3389/fpls.2024.1367121)
Supplement: Supplementary Table 6 — Comparison of differential hormones [file Table_6.pdf]

Table S6 Comparison of differential hormones

group name	All sig diff	up regulated	down regulated
Tub19 vs CK6	31	8	23
Tub6 vs CK6	15	4	11
Tub19 vs Tub6	26	10	16
