# Supplementary material for: Formation mechanism and regulation analysis of trumpet leaf in Ginkgo biloba L
Source: Front Plant Sci. 2024 Jul 17;15:1367121. doi: 10.3389/fpls.2024.1367121 (PMC11288918; doi:10.3389/fpls.2024.1367121)
Supplement: Supplementary Table 7 — The expression of 7 differential metabolites screened out [file Table_7.pdf]

**Table S7** he expression of 7 differential metabolites screened out

| **Compounds** | **Tub6** | **Tub19** | **CK6** |
|---|---|---|---|
| meta-Topolin | 0.1136±0.0050 | 0.2255±0.0331 | 0±0 |
| 2-Methylthio-cis-zeatin | 0.0936±0.0082 | 0.0697±0.0075 | 0±0 |
| Gibberellin A4 | 0±0 | 0±0 | 0.6954±0.0771 |
| Gibberellin A15 | 0±0 | 0±0 | 0.1190±0.0044 |
| 3-oxo-2-(2-(Z)-Pentenyl) cyclopentane-1-butyric acid | 18.1343±1.6699 | 22.3201±3.4101 | 4.6836±0.4061 |
| cis(+)-12-Oxophytodienoic acid | 173.4192±27.8129 | 90.7727±22.8969 | 17.5120±2.5591 |
| (±) Strigol | 76.2878±4.1616 | 41.4903±2.0932 | 0±0 |
