# Supplementary material for: Formation mechanism and regulation analysis of trumpet leaf in Ginkgo biloba L
Source: Front Plant Sci. 2024 Jul 17;15:1367121. doi: 10.3389/fpls.2024.1367121 (PMC11288918; doi:10.3389/fpls.2024.1367121)
Supplement: Supplementary Table 8 — Statistical table of differential metabolites [file Table_8.pdf]

Table S8 statistical table of differential metabolites

Class I	Number	Compounds	kegg_map
Phenolic acids	14	Salidroside、1-O-Salicyloyl-β-D-glucose、3,4-Dihydroxybenzoic acid (Protocatechuic acid)*、Bilobol、Gallic acid-4-O-glucoside、Protocatechuic acid-4-O-glucoside、Vanilloloside、Ethyl cinnamate、3,4-dihydroxyphenylethanol-β-D-glucopyranoside、Glucosyloxybenzoic acid、2,4-Dihydroxybenzoic acid、4'-Hydroxy-3'-methoxyacetophenone (Acetovanillone)、5-O-Feruloyl quinic acid glucoside*、	ko00350,ko01100,ko01110
Flavonoids	16	5,2'-Dihydroxy-7-methoxyflavanone、Tectochrysin 5,4'-Dihydroxy-7-methoxyflavanone (Sakuranetin)、5-Hydroxy-3,7,3',4'-tetramethoxyflavone (Retusin)、5,4'-Dihydroxy-3,7-dimethoxyflavone(Kumatakenin)、Kaempferide-3-O-(6''-malonyl)glucoside、Monohydroxy-hexamethoxyflavone、7-O-Methylnaringenin、Hydroxygenkwanin、4',5-Dihydroxy-3',5'-dimethoxyflavone、3,7-Di-O-methylquercetin、Eriodictyol (5,7,3',4'-Tetrahydroxyflavanone)、Quercetin-3-O-(2''-O-acetyl)glucuronide、Eupatilin (5,7-Dihydroxy-3',4',6-Trimethoxyflavone)*、Eupatorin; 3',5-Dihydroxy-4',6,7-Trimethoxyflavone*、Mearnsetin	ko00941,ko01100 ,ko00944,ko01110
Lignans	2	Piperitol、Matairesinol-4,4'-di-O-glucoside	-
Tannins	1	Procyanidin B3	-
Terpenoids	2	3-Hydroxyolean-12-en-28-oic acid (Oleanolic acid)、3-Hydroxyurs-12-en-28-oic acid (Ursolic acid)	-
Organic acids	1	3-Methylmalic acid*	ko00290,ko00660,ko01100, ko01210,ko01230
Lipids	4	3-Hydroxyoctadecanoic Acid、20-Carboxyarachidonic Acid、Hydroxyicosanoic Acid、Docosanoic acid (Behenic acid)	ko00073,ko01040
