# Supplementary material for: Formation mechanism and regulation analysis of trumpet leaf in Ginkgo biloba L
Source: Front Plant Sci. 2024 Jul 17;15:1367121. doi: 10.3389/fpls.2024.1367121 (PMC11288918; doi:10.3389/fpls.2024.1367121)
Supplement: Supplementary file 9 [file DataSheet_1.pdf]

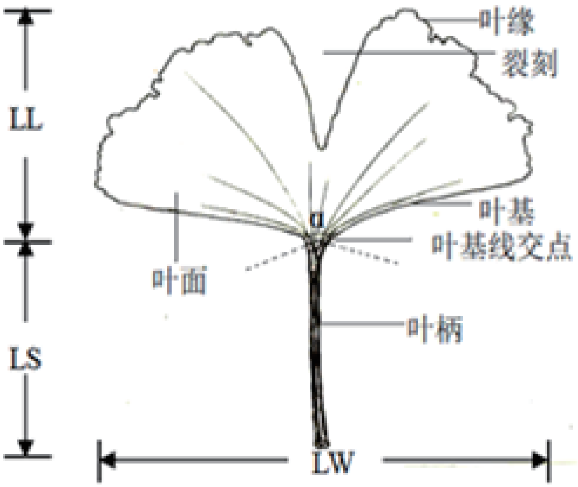

Figue S1 Phenotypic data measurement diagram. L-L is leaf length ; L-W is leaf width ; L-S is petiole length ; α is the baseline angle.

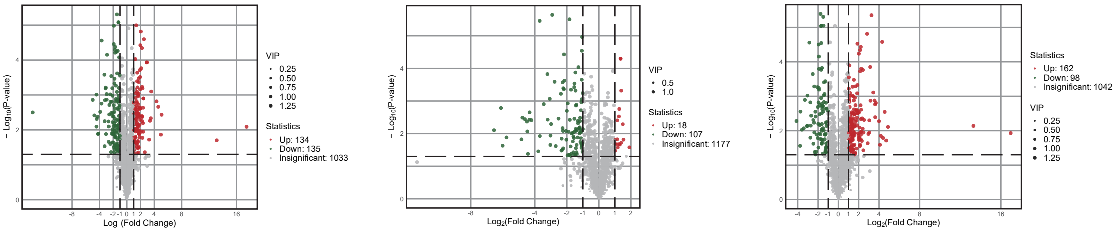

Tub19 vs CK6 Tub6 vs CK6 Tub19 vs Tub6

Figue S2 Volcano diagram of differential metabolites of *G. biloba* in each comparison group. Each point in the volcano map represents a metabolite, where the green point represents a down-regulated differential metabolite, the red point represents an up-regulated differential metabolite, and the gray represents a metabolite that is detected but not significantly different ; the abscissa represents the logarithm of the relative content difference multiple of a metabolite in the two groups of samples (log_2FC). The larger the absolute value of the abscissa, the greater the relative content difference of the substance between the two groups of samples. The ordinate represents the difference significance level ($-log_{10}$P-value), and the size of the dots represents the VIP value.

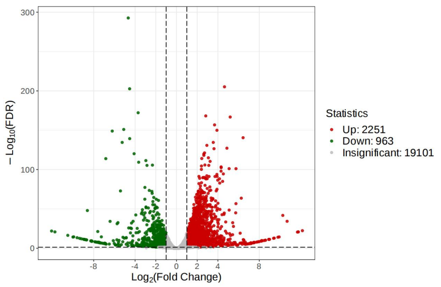

Tub19 VS CK6

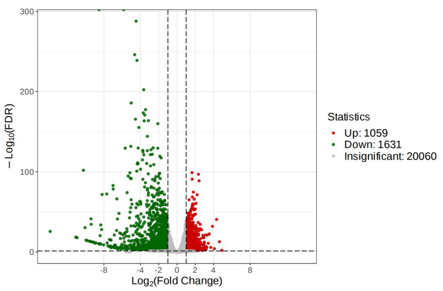

Tub6 VS CK6

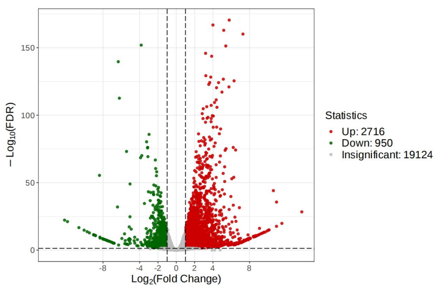

Tub19 VS Tub6

Figue S3 Differentiallyexpressedgenevolcanomapofeachcomparisongroup.The abscissarepres entsthech angeofgeneexpressionmultiples,andtheordinate representsthesignificancelevelofdiff erentialgenes.There ddotsrepresent up-regulateddifferentialgenes,thegreendotsrepresentdown -regulateddifferential genes,an dthegraydotsrepresentnon-differentiallyexpressedgenes.

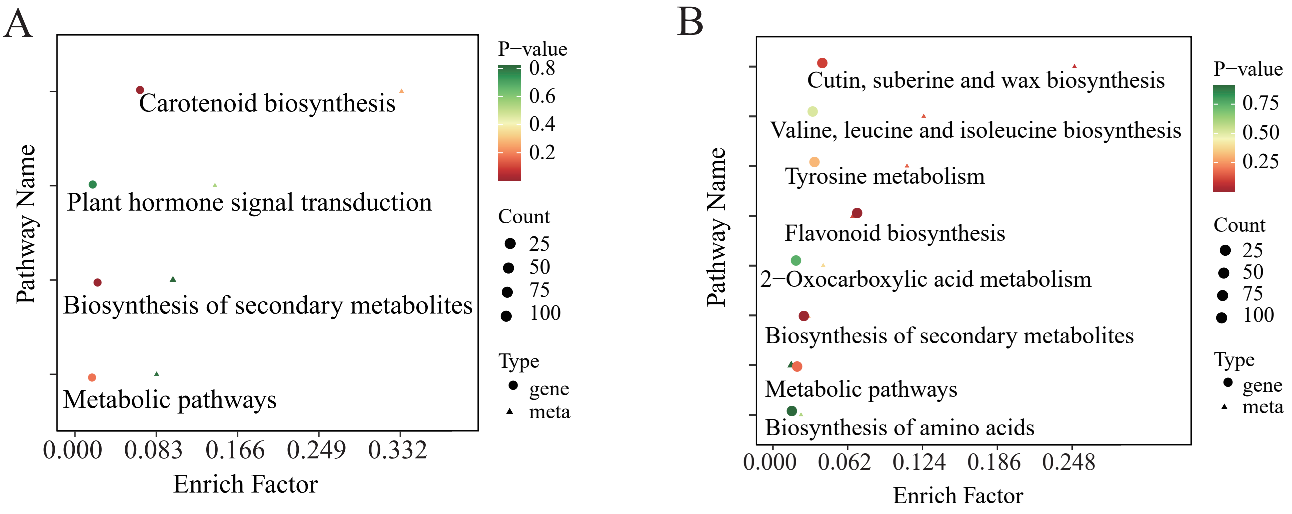

Figure S4 Joint analysis of differential hormones, metabolites and differential genes. **(A)** Differential gene and hormone common pathway map. **(B)** Differential gene and hormone common pathway map.
